# Intake of Lipid-based Nutrient Supplements during Illness and Convalescence among Moderately-underweight Malawian Children

**Published:** 2008-12

**Authors:** Valerie L. Flax, Kenneth Maleta, Ulla Ashorn, Mark J. Manary, André Briend, Per Ashorn

**Affiliations:** 1 School of Public Health, University of Tampere, Finland; 2 Department of International Health, University of Tampere Medical School, Finland; 3 College of Medicine, University of Malawi, Blantyre, Malawi; 4 Washington University School of Medicine, St. Louis, USA; 5 IRD, Département Sociétés et Santé, Paris, France; 6 Department of Paediatrics, University of Tampere Hospital, Finland

Sir,

The effect of infections and other illnesses on the nutritional status of a child depends on the type and severity of the illness, initial nutritional status of the individual, and food intake during illness and convalescence (1). In practice, illnesses in children are often associated with reduced appetite and decreased food intake (2,3). Although short-term nutritional losses can be overcome (4), illnesses predispose children in resource-poor settings to weight loss and development of malnutrition (2).

Lipid-based nutrient supplements (LNS) are ready-to-use foods that have been used for treating children with severe acute malnutrition and for preventing malnutrition and linear growth failure (5,6). Because of their high energy and nutrient content, LNS could be used for supplementation during and soon after illness to mitigate the impact of illness-associated anorexia on the nutritional status of children. To date, little is known about the acceptability of LNS during and after common childhood illnesses. This longitudinal study provides preliminary comparative information on LNS intake by children on ill, convalescent and healthy days.

The study was conducted around Lungwena and Malindi, a rural area in southern Malawi.

Subjects were 16 moderately-underweight (weight-for-age *z*-score <-2) children aged 6-17 months participating in a study on the incorporation of LNS into home diets. Details of the selection criteria are published elsewhere (7).

During the 12 weeks of supplementation, research assistants delivered three 250-g plastic jars of LNS to homes of the participants fortnightly. They visited the study families five evenings per week to weigh LNS jars and used a semi-structured questionnaire to obtain information on child morbidity. Caregivers were advised to feed the child seven teaspoons (≈50 g) of LNS per day. The LNS, produced in Blantyre, Malawi, was made of peanut butter, milk powder, sugar, oil, and vitamin/mineral mix.

The analysis initially compared LNS intake on days when children were reported to be ill versus not ill. The categories ‘ill’ and ‘not ill’ were then further subdivided. Mutually-exclusive groups were developed to categorize illnesses: fever was given the first priority in forming the groups, followed by diarrhoea, as these have the largest effect on food intake of children (2,3). Fever was defined as fever only or in combination with any other symptom; diarrhoea was defined as diarrhoea only or in combination with any symptom other than fever; cough was defined as cough only or cough together with ‘other’ illnesses; and ‘other’ illnesses contained only symptoms in the ‘other’ category, including sores, pus from ears, vomiting, and prolonged crying.

Days when children were reported ‘not ill’ were coded into three categories: early convalescence (day 1-7 following illness), late convalescence (day 8-14 following illness), and healthy (other days). Convalescent days were coded starting from the first day the child was not reported to be ill and continuing until the next reported illness (if any). The first days of data collection were omitted for children who were reported to be ‘not ill’ either until they were reported to be ill or until the eighth day, at which point they were counted as healthy. When no information was available, the health status of the child was assumed to be identical to the first day when data were available again.

Linear regression analysis was used for calculating the mean LNS intake and to test for the difference in LNS intake on ‘ill’ versus ‘not ill’ days. Means and confidence intervals for LNS intake were adjusted for within-subject correlation by the Huber-White robust standard error.

At enrollment, the mean (standard deviation [SD]) age of the participants was 13 (3) months, the mean (SD) weight-for-age *z*-score was −2.6 (0.3), the height-for-age *z*-score was −1.8 (0.7), and the weight-for-height *z*-score was −1.2 (0.9). The children lived in households with a mean (SD) of 5 (2) members, and their median birth-order was 3.

Data were collected from all the 16 participants during the first eight weeks that LNS was provided. One child moved away after the eighth week, and the remaining children provided information during the final four weeks of follow-up. In total, 845 days of data were collected (63% of the days when LNS was available). Thirteen (1.5%) of these days were excluded from the analysis because the daily measurements were suggestive of food-sharing (LNS use >150 g/day).

Illness was reported on 142 (17%) days. Of these days, 57 (40%) were classified as fever, 30 (21%) as diarrhoea, 44 (31%) as cough, and 11 (8%) as other illness days. Fifteen children were ill on at least one day; the child who moved away during the study had no reported illness.

The mean LNS intake was 43.4 g (≈230 kcal) on ‘ill’ and 50.6 g (≈268 kcal) on ‘not ill’ days (difference=7.2 g, 95% CI 0.6-13.8 g, p<0.034). The mean consumption of LNS for different types of illness is shown in the table. Compared to days when children were not reported ill, the mean daily LNS intake was 14% (≈38 kcal) lower on ill days, ranging from <1% (≈2 kcal) on ‘other’ illness days to 25% (≈65 kcal) on fever days. On an individual level, 12 of 15 participants had a higher mean LNS intake on days when they were not reported to be ill than on those when they were.

Of the 690 days when children were reported to be ‘not ill', 32 days were omitted from the analysis of convalescent and healthy days because the timing of the last illness before the study began was unknown. During a two-week period of convalescence from illness, consumption of LNS was similar to the mean for healthy days (Table).

**Table.** Quantity of LNS consumed daily by health status

Health status	Mean (g)	95% CI	1st quartile	3rd quartile
Days when reported ill				
Fever (n=10)	38.2	26.8, 49.6	20.0	52.5
Diarrhoea (n=9)	45.6	33.6, 57.6	29.8	64.0
Cough (n=11)	47.0	37.8, 56.2	27.5	58.8
Other (n=6)	50.5	35.1, 65.8	36.0	76.0
Days when reported not ill				
Early convalescence (n=15) (1-7 day(s) after illness)	50.4	46.8, 54.1	29.0	65.0
Late convalescence (n=150 (8-14 days after illness)	52.3	49.1, 55.5	34.0	69.3
Healthy (n=16) (>14 days after illness)	49.6	47.1, 52.0	31.0	64.5

CI=Confidence Interval; LNS=Lipid-based nutrient supplement

Findings of this preliminary study suggest that young children readily eat LNS during illness and convalescence. The accuracy of measuring child illness and the use of supplementary food are two possible limitations of the study, but these are unlikely to affect the internal validity of or the general picture obtained from these data. Maternal reports of child illness in low-income countries have been validated, even for two-week periods (8). This study minimized caregiver's recall bias by asking about a 24-hour period. Some food-sharing may have occurred within households. However, the pattern of lower LNS intake during illness, with a larger reduction in intake during fever, is similar to the pattern of food intake found in other studies of sick children (2,3) and suggests that most of supplement was consumed by the intended beneficiaries.

The World Health Organization recommends that caregivers continue to feed children during illness and increase intake thereafter (9). Identifying foods or supplements, such as LNS, that underweight children accept when they are ill or recovering and that are easy to use, may help caregivers follow feeding recommendations and prevent a worsening of the nutritional status of children (1,2). The results of this small pilot study suggest that LNS is well-accepted by ill and convalescing children and, as such, might offer the possibility for short-term feeding interventions. This hypothesis could be tested in a randomized trial where young children are allocated to receive supplementary LNS regularly over a defined age-range or as 2-3-week auxiliary treatment during and after acute illnesses.

## ACKNOWLEDGEMENTS

The authors are grateful to Yin Bun Cheung for statistical advice.
